# Antimicrobial Peptide Papiliocin–Carbon Nanotube Hybrids: Potential Dual-Action Agents for Antimicrobial Activity and Apoptotic Cancer Cell Death

**DOI:** 10.3390/molecules31101715

**Published:** 2026-05-18

**Authors:** Konstantinos Zacheilas, Myrto Margariti, Maria Apostolia Pissia, Rigini M. Papi

**Affiliations:** 1 Laboratory of Biochemistry, Department of Chemistry, Aristotle University of Thessaloniki, 54124 Thessaloniki, Greece; 2 Laboratory of Food Chemistry and Technology, Department of Chemistry, Aristotle University of Thessaloniki, 54124 Thessaloniki, Greece

**Keywords:** antimicrobial peptides, papiliocin, carbon nanotubes, delivery system, microbial resistance, *Escherichia coli*, *Staphylococcus aureus*, *Candida albicans*, HeLa, HaCat, cytotoxicity, apoptosis

## Abstract

The emerging threat of antibiotic-resistant pathogens and the limitations that conventional cancer chemotherapies display have created an urgent need for the development of innovative therapeutic strategies. Combining the pleiotropic biological roles of antimicrobial peptides (AMPs) and nanomaterials through their conjugation presents a promising possibility of targeting both microbial membranes and malignant cells. In the present study, we engineered a novel bioactive material by immobilizing the insect-derived AMP Papiliocin onto multi-walled—decorated with polyethylene–glycol—carbon nanotubes (PEG-MWCNTs) to prevent proteolytic degradation of the peptide and enhance its cellular delivery. Recombinant Papiliocin was cloned, heterologously expressed, purified and conjugated onto the PEG-MWCNT carrier. Successful expression and conjugation were validated via immunoblotting and Fourier transform infrared (FT-IR) spectroscopy, respectively. Further physicochemical characterization of the bionanocomposites was conducted using Dynamic Light Scattering (DLS) and Zeta potential measurements. Biologically, the biofunctionalized material exhibited potent, broad-spectrum antimicrobial activity both on *Staphylococcus aureus* and *Escherichia coli*, inhibiting almost 90% of the latter’s growth, highlighting the bioconjugate’s specific interactions with the Gram-negative pathogens’ membranes. Furthermore, it significantly reduced biofilm formation in *Candida albicans*, as indicated by the TCP assay. In parallel with its antimicrobial effects, CNTs-PEG–Papiliocin significantly reduced cancer cell viability and induced apoptosis via the extrinsic apoptosis pathway in HeLa cells, a response assisted by efficient intracellular delivery. Notably, cytotoxicity assays demonstrated lesser cytotoxic effect against non-tumorigenic HaCaT cells relative to the cancerous cell line. Collectively, these findings indicate the Papiliocin–biofunctionalized CNTs as a versatile, dual-action therapeutic agent with potential for antimicrobial activity and anticancer mode of action.

## 1. Introduction

Today’s biomedical landscape is being dominated by two escalating global health crises; one of them being the rapid proliferation of multidrug resistant pathogens while the other concerns the limitations and challenges of cancer therapy. A new “post-antibiotic era” is near due to the misuse of conventional antibiotic which drove the evolution of impervious-to-treatment superbugs, leading to prolonged illnesses, higher mortality rates and a staggering economic burden on healthcare systems worldwide [1,2]. Concurrently, the limitations and challenges that traditional chemotherapy displays include indiscriminate attacks on dividing cells while manifesting damage to healthy tissues [3]. Furthermore, the clinical efficacy of many agents is frequently compromised by the development of multidrug resistance (MDR) mechanisms, such as efflux pumps [4]. These persistent challenges underscore the need for targeted delivery systems to improve intracellular localization.

Both these problems have created an urgent need that drive the discovery of promising bioactive molecules. Interestingly, antimicrobial peptides (AMPs), biomolecules acting as “first-line-of-defense” agents of the innate immune system of various organisms, have emerged as a multi-faceted solution [5,6]; unlike traditional antibiotics that target specific intracellular enzymes, AMPs, primarily cationic AMPs, function by electrostatically disrupting the integrity of negatively charged cell membranes [7]. Both bacterial and cancer cell membranes tend to have a net negative charge due to the presence of anionic components such as lipopolysaccharides and phosphatidylserine, respectively, making them ideal selective targets for AMPs [8,9]. Papiliocin, a 37-residue cecropin-like peptide isolated from the larvae of the swallowtail butterfly (*Papilio xuthus*), has garnered scientific attention among the wide variety of AMPs found [10]. It exhibits 78.4% homology with Cecropin A, a peptide known for its antimicrobial and anticancer properties [11,12,13]. The insect-derived peptide has an amphipathic α-helical structure, consisting of a highly cationic N-terminal helix which pulls the peptide toward the anionic surface, a flexible hinge region, and a hydrophobic C-terminal helix [14,15]. Papiliocin exhibits broad-spectrum antimicrobial activity, but its potency lies mainly against Gram-negative strains, with MIC values ranging from 0.25 to 1 μM. In contrast, activity against Gram-positive *S. aureus* was significantly lower with an MIC of 32 μM, confirming the peptide’s selectivity for LPS-rich anionic membranes [10]. The peptide’s activity is not confined to bacteria only, Papiliocin also inhibits fungi growth and causes apoptosis to yeast cells [16]. Furthermore, it displays a high therapeutic index due to its immunomodulatory role by competitively blocking the TLR4 signaling pathway, thus preventing endotoxin-mediated sepsis-induced cytokine storms while presenting low to no cytotoxicity against macrophages and no hemolytic activity in concentrations under 100 μM [10,17].

However, intrinsic pharmacological barriers impede the clinical translation of AMPs [18]. The most critical one being their low metabolic stability, which makes them susceptible to rapid degradation by proteolytic enzymes, known as proteases, found in the gastrointestinal tract and blood plasma, resulting in incredibly short in vivo half-lives [19]. Therefore, creating a delivery system that can stabilize the peptide, prevent its premature breakdown before it reaches the target, and facilitate its cellular accumulation more effectively is the primary challenge. Nanotechnology offers a sophisticated solution to these delivery hurdles [20]. Carbon nanotubes (CNTs), cylindrical nanostructures composed of rolled graphene sheets, have established themselves as effective components [21]. The high surface-area-to-volume ratio of these nanomaterials enables high-density loading of therapeutic cargo [22]. A key benefit of CNTs is their ability to breach cellular barriers. This is achieved through multiple internalization mechanisms: while primarily driven by endocytosis and micropinocytosis, CNTs can also undergo passive, non-energy-dependent diffusion across the lipid bilayer [23]. Due to these properties, a great deal of research has used CNTs as adaptable nanocarriers, functionalizing their high-surface-area scaffolds with a wide range of bioactive molecules such as DNA [24], therapeutic proteins [25], chemotherapeutic agents [26] and a variety of other therapeutic molecules [27,28,29] to guarantee site-specific accumulation and improved intracellular uptake. Not only that, but CNTs can exhibit antimicrobial effects by interacting with bacterial surfaces and inducing oxidative stress through the generation of Reactive Oxygen Species (ROS), leading to lipid peroxidation and depletion of intracellular antioxidant reserves [30]. Therefore, conjugating peptide-based therapeutics to the surface of carbon nanotube scaffolds, sterically protects peptides from proteolytic enzymes and overcomes poor bioavailability associated with free peptide administration, significantly extending their circulation time.

Therefore, the primary objective of this study is to design, synthesize and evaluate a novel therapeutic nanohybrid that addresses the critical limitations of peptide-based therapy: production cost, stability and delivery. To achieve this, we first implement a cost-effective biotechnological approach via heterologous expression to produce recombinant Papiliocin, circumventing the economic constraints of traditional chemical synthesis. Subsequently, the peptide is conjugated to carbon nanotubes (CNTs) to engineer a stable “dual-action” agent. The specific aims are to validate the hybrid’s potent antimicrobial efficacy against *E. coli* and *S. aureus*, and to assess its ability to eradicate resistant *C. albicans* biofilms. Simultaneously, another axis of our work lies in understanding the intracellular dynamics and anticancer mode of action of the hybrid. Using immunofluorescence microscopy, we monitored the spaciotemporal trafficking of the CNTs–Papiliocin system migrating from a dispersed nuclear and cytoplasmic distribution at 24 h to a predominant nuclear accumulation at 48 h. This exact translocation of Papiliocin targeting the nucleus that is hypothesized to drive the extrinsic apoptotic pathway will be thoroughly mapped by identifying Caspase-8 activation and the subsequent cleavage of Poly (ADP-ribose) polymerase 1 (PARP1). The efficacy seen in all these assays highlights the Papiliocin–CNT hybrid’s biological potential.

## 2. Results

### 2.1. Cloning, Expression and Purification of Papiliocin

Papiliocin coding sequence was successfully PCR-amplified from the pEX-*Papiliocin* template using primers containing NdeI and HindIII restriction sites. The amplified product was ligated, using a T4 DNA ligase, into the pET-29c(+) vector to generate the expression construct. The ligation mixture was used to transform *E. coli* TOP10F-competent cells, which were further employed to isolate the constructed plasmid (pET29c-*Papiliocin*). Agarose gel electrophoresis confirmed the integrity of the gene insert and the successful generation of the pET-*Papiliocin* vector (Figure S1).

To produce the peptide, the pET29c–*Papiliocin* construct was transformed into *E. coli* Rosetta (DE3) cells and overexpression of Papiliocin, as a C-terminal dodecahistidine fusion protein, in TB medium was performed. Following expression, the peptide was purified via Ni-TED affinity chromatography and isolated using Amicon Ultra Centrifugal Filters. SDS-PAGE analysis visualized by silver staining revealed a pure peptide band at the expected molecular weight. The identity of the purified product was confirmed via immunoblotting with a mouse monoclonal anti-His-tag antibody (Figure S2).

### 2.2. Immobilization of Papiliocin onto Carbon Nanotubes and Bionanocomposite Characterization

The immobilization of recombinant Papiliocin onto carbon nanotubes (CNTs) was designed to achieve three primary objectives: (i) to facilitate stable cellular uptake while retaining peptide activity; (ii) to stabilize the peptide and protect it from proteolytic degradation; and (iii) to leverage the combinatorial antibacterial effects of both the CNTs and the peptide. As previously described [25], oxidized CNTs (9.5 nm diameter × 1.5 nm length) were selected as the carrier due to their high aspect ratio and ability to penetrate cell membranes. In order to enhance the CNTs’ hydrophilicity and biocompatibility, they were surface-modified with bis(3-aminopropyl)polyethylene glycol (PEG). This was achieved via a two-step activation of the CNT carboxyl groups using EDC and NHS, followed by reaction with the amino groups of the PEG linker. Papiliocin was subsequently conjugated to the PEGylated CNTs via a second activation step. Following the reaction, unconjugated protein was removed via filtration, and Bradford assay was used to determine any unconjugated protein. The final functionalized constructs were analyzed by FT-IR to verify successful peptide immobilization.

**Figure 1:**
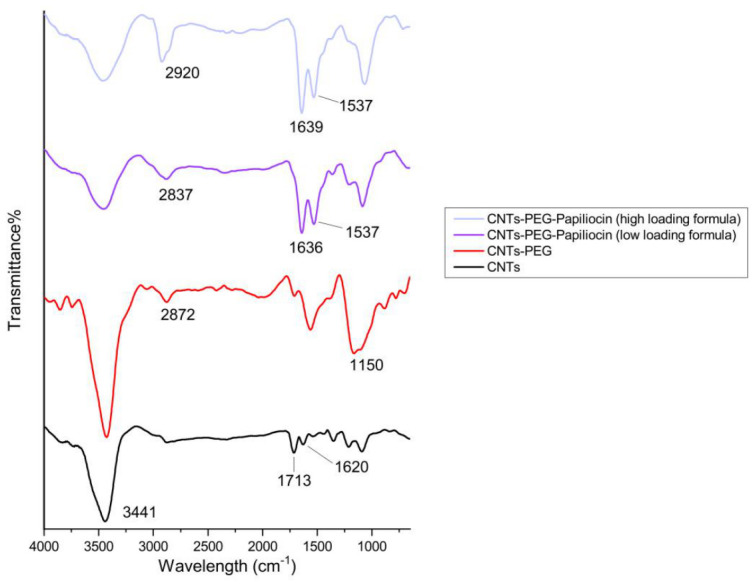
FTIR spectra of functionalized carbon nanotubes. Characterization of pristine CNTs (black), PEG-functionalized CNTs (red) and Papiliocin-loaded PEGylated CNTs at low-loading (purple) and high-loading (ice blue) concentrations.

As shown in Figure 1, the spectrum of the starting material (oxidized CNTs—carboxylic acid functionalized) confirmed the presence of oxygen-containing groups, with a broad -OH stretching band at 3441 cm^−1^ and a distinct C=O stretching peak at 1721 cm^−1^, characteristic of the pre-existing carboxylic acid groups on the oxidized nanotubes. Additionally, the graphitic backbone was identified by the C=C stretching vibration at 1620 cm^−1^. Following PEGylation (CNTs-PEG), the successful attachment of PEG chains was evidenced by the emergence of the characteristic C-O-C ether stretching at 1150 cm^−1^ and the alkyl C-H signals at 2872 cm^−1^. For the CNTs-PEG–Papiliocin complexes, the incorporation of the peptide was clearly demonstrated by the appearance of the Amide I band at 1636–1639 cm^−1^ (C=O stretching) and the Amide II band at 1537 cm^−1^ (N-H bending). The presence of the peptide was further supported by a noticeable shoulder in the 3400–3500 cm^−1^ region, attributed to N-H stretching vibrations overlapping with the hydroxyl band. Notably, the high-loading formula exhibited significantly more intense amide peaks compared to the low-loading formula, confirming a concentration-dependent loading of Papiliocin onto the functionalized CNT platform.

Aside from FT-IR, DLS and zeta potential measurements were performed to further characterize the nanocomposites (detailed data provided in Supplementary Materials—Figures S3 and S4). DLS analysis in PBS showed a concentration-dependent increase in the hydrodynamic diameter, with peaks shifting from approximately 250 nm for CNT-PEG to 350 nm for the high-loading formula. This size increment was accompanied by a significant shift in surface charge; zeta potential measurements revealed that the carrier’s initial negative charge was neutralized toward near-neutral values as peptide loading increased. These results confirm that the high-loading formulation achieves a higher surface density of Papiliocin.

### 2.3. Papiliocin-Biofunctionalized Carbon Nanotubes Significantly Inhibit the Growth of Both Gram-Negative and Gram-Positive Bacteria

**Figure 2:**
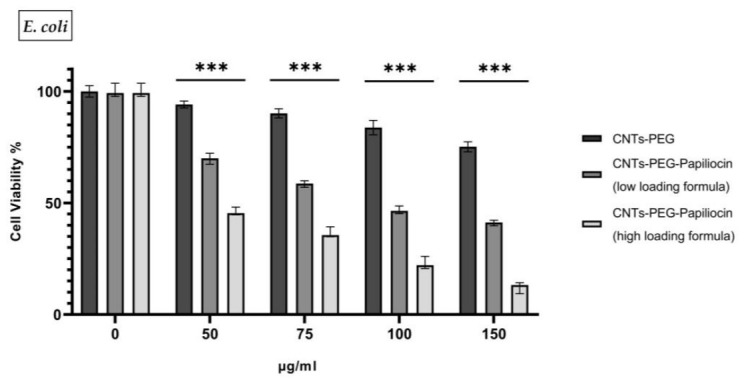
In vitro antibacterial activity of Papiliocin–biofunctionalized carbon nanotubes (CNTs). The cell viability of *E. coli* was assessed after 24 h of incubation with CNTs-PEG (control), CNTs-PEG–Papiliocin (low-loading formula) and CNTs-PEG–Papiliocin (high-loading formula) at concentrations of 0, 50, 75, 100, and 150 μg/mL. Statistical analysis was performed using GraphPad Prism software (Version 8.0.1) (GraphPad, San Diego, CA, USA) Statistical significance was determined using the *p*-value (*p* < 0.001 (represented by ***)).

**Figure 3:**
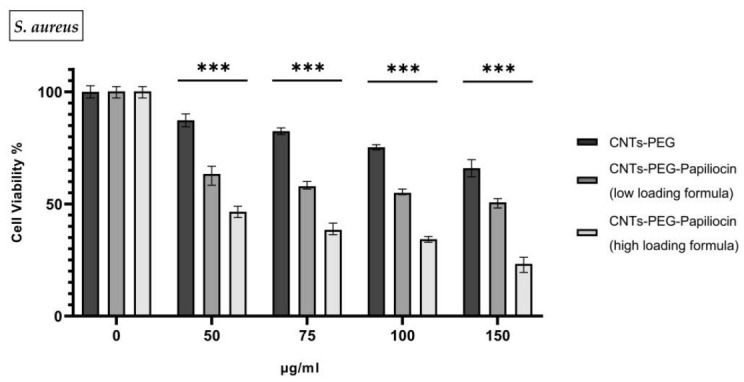
In vitro antibacterial activity of Papiliocin–biofunctionalized carbon nanotubes (CNTs). The cell viability of S. aureus was assessed after 24 h of incubation with CNTs-PEG (control), CNTs-PEG–Papiliocin (low-loading formula) and CNTs-PEG–Papiliocin (high-loading formula) at concentrations of 0, 50, 75, 100 and 150 μg/mL. Statistical analysis was performed using GraphPad Prism software (Version 8.0.1) (GraphPad, San Diego, CA, USA). Statistical significance was de-termined using the *p*-value (*p* < 0.001 (represented by ***)).

The antibacterial potential of Papiliocin–biofunctionalized carbon nanotubes (CNTs) was evaluated against Gram-negative *E. coli* and Gram-positive *S. aureus*. As illustrated in Figure 2 and Figure 3, the biofunctionalized nanocomposites demonstrated significant, dose-dependent growth inhibition across both bacterial strains compared to the control group (CNTs-PEG), indicating broad-spectrum antibacterial activity.

Distinct immobilization ratios—a high-loading formulation and a lower-loading formulation—were selected to determine the optimal biofunctional threshold. Since antimicrobial peptides are known to be highly bioactive even at low concentrations, the low-loading ratio was employed to assess whether a reduced peptide density could sustain bactericidal efficacy, or if a higher saturation was required to overcome bacterial resistance mechanisms.

For both bacterial strains, the pristine CNTs-PEG vehicle exhibited mild cytotoxicity, maintaining cell viability above 60–75% even at the highest concentration of 150 µg/mL. However, the conjugation of Papiliocin significantly potentiated the antimicrobial effect. The high-loading formulation consistently outperformed the low-loading formulation, suggesting that while the peptide is active at lower densities, a higher surface coverage is critical for maximizing efficiency. Specifically, at 150 µg/mL, the high-loading formulation reduced *E. coli* viability to approximately 13% and *S. aureus* viability to roughly 23%. It is important to annotate that *S. aureus* seems to be more susceptible to the CNTs-PEG vehicle control sample opposed to *E. coli*.

This enhanced potency was further quantified by determining the half-maximal inhibitory concentration (IC_50_). For *E. coli*, the IC_50_ decreased dramatically from 215.6 µg/mL (CNTs-PEG) to 110.2 µg/mL (low loading) and finally to 58.49 µg/mL for the high-loading formulation. A similar trend was observed for *S. aureus*, with IC_50_ values of 189.2 µg/mL (CNTs-PEG), 126.1 µg/mL (low loading), and 65.47 µg/mL (high-loading). These values confirm that increasing the peptide load significantly lowers the concentration required to achieve bactericidal activity.

The pronounced susceptibility of the bacteria, particularly the Gram-negative strain, aligns with the inherent properties of Papiliocin. As a cationic antimicrobial peptide, Papiliocin targets the anionic components of bacterial membranes via electrostatic attraction. This interaction is particularly effective against Gram-negative bacteria like *E. coli*, where the outer membrane is rich in negatively charged lipopolysaccharides (LPSs). The high density of positive charges on the higher loaded biofunctionalized CNTs likely facilitates a rapid and strong binding to these negative surface charges, leading to membrane destabilization and cell death.

These results confirm a potent joint activity where the CNTs serve as a high-surface-area scaffold that concentrates the cationic peptide directly onto the bacterial surface, significantly enhancing the bactericidal outcome (*p* < 0.001%) compared to the control.

### 2.4. Papiliocin–Carbon Nanotubes Conjugates Reduce Candida albicans Biofilm Formation

**Figure 4:**
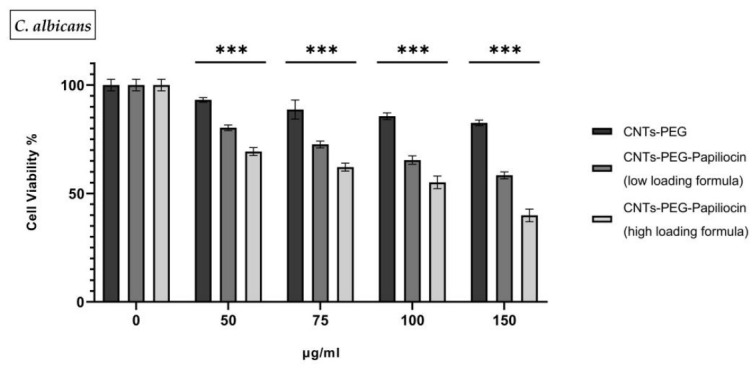
*C. albicans* biofilm viability following treatment with functionalized CNTs. Biofilms were treated with varying concentrations (50–150 μg/mL) of CNTs-PEG alone and two ratios of Papiliocin-conjugated CNTs (low- and high-loading formulas). Statistical analysis was performed using GraphPad Prism software (Version 8.0.1) (GraphPad, San Diego, CA, USA). Statistical significance was determined using the *p*-value (*p* < 0.001 (represented by ***)).

To evaluate the potential of the bionanocomposites to combat resistant fungal biofilms, a TCP assay was performed against *C. albicans*. As shown in Figure 4, the CNTs-PEG control exhibited negligible antibiofilm activity, retaining more than 82% viability even at the highest tested concentration (150 µg/mL).

In contrast, the biofunctionalization with Papiliocin significantly enhanced the system’s ability to disrupt the biofilm. Notably, even at the lowest conjugation ratio, the nanocomposites demonstrated statistically significant activity (*p* < 0.001%) compared to the control group. However, the extent of biofilm eradication was directly correlated with the peptide payload: the more loaded the system was with Papiliocin, the more effectively it reduced the biofilm mass.

Specifically, while the low-loading formulation reduced biofilm viability to approximately 60% at 150 µg/mL, the high-density formulation achieved a much sharper decline, dropping viability to 39.9%. This indicates that while the presence of Papiliocin is sufficient to initiate antibiofilm activity, a critical density of the peptide is required to penetrate the Extracellular Polymeric Substance (EPS) matrix and maximize fungal cell death. Forthe control group and the lower conjugation ratio (low-loading formula), the IC_50_ values could not be determined, as the biofilm viability did not drop below 50% within the tested concentration range. Conversely, the superior efficacy of the high-loading formulation allowed for a distinct IC_50_ determination of 114.8 µg/mL, marking it as the only formulation capable of achieving therapeutic inhibition against the fungal biofilm matrix.

### 2.5. Cytotoxicity of CNTs-PEG–Papiliocin System

Based on the superior bioactivity observed in our antimicrobial assays, the CNTs-PEG–Papiliocin (high loading) conjugate was selected as the optimal formulation for further evaluation in mammalian cells. The cytotoxic potential of this system against HeLa cancer cells was assessed using the MTT assay after 24 and 48 h of incubation. To verify that the observed cytotoxicity was attributed specifically to the antimicrobial peptide and not to the nanocarrier or the functionalization process, CNTs-PEG and CNTs-PEG-GFP were employed as controls.

**Figure 5:**
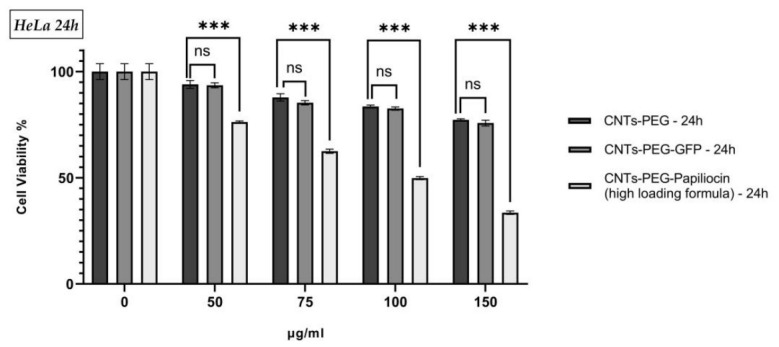
In vitro cytotoxicity of CNTs-PEG, CNTs-PEG-GFP and CNTs-PEG–Papiliocin (high-loading formula) on HeLa cells determined by MTT assay. The cells were treated with various concentrations (0–150) for 24 h. Statistical analysis was performed using GraphPad Prism software (Version 8.0.1) (GraphPad, San Diego, CA, USA). Statistical significance was determined using the *p*-value (*p* < 0.001 (represented by ***)—ns stands for no statisticl significance).

**Figure 6:**
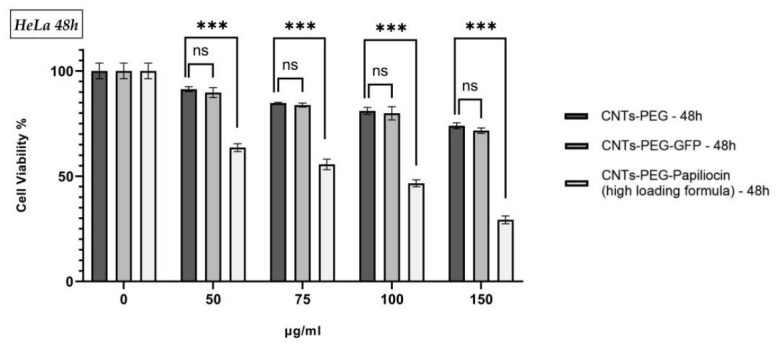
In vitro cytotoxicity of CNTs-PEG, CNTs-PEG-GFP and CNTs-PEG–Papiliocin (high-loading formula) on HeLa cells determined by MTT assay. The cells were treated with various concentrations (0–150) for 48 h. Statistical analysis was performed using GraphPad Prism software (Version 8.0.1) (GraphPad, San Diego, CA, USA). Statistical significance was determined using the *p*-value (*p* < 0.001 (represented by ***)—ns stands for no statisticl significance).

As presented in Figure 5 and Figure 6, the cell viability of HeLa cells treated with the control groups (CNTs-PEG and CNTs-PEG-GFP) remained relatively high (>70%) even at the highest concentration of 150 μg/mL. Notably, no statistically significant difference (ns) was observed between the CNTs-PEG and CNTs-PEG-GFP groups at any concentration or time point, confirming the biocompatibility of the carrier system. In contrast, the selected CNTs-PEG–Papiliocin (high-loading formula) system exhibited a marked, dose-dependent reduction in cancer cell viability. At concentrations of 100 μg/mL and above, the viability of cells treated with the Papiliocin conjugate was significantly lower (*p* < 0.001) compared to both control groups. At the maximum concentration of 150 μg/mL cell viability dropped to approximately 35% and 30% after 24 and 48 h, respectively. These results confirm that the potent bioactivity of the Papiliocin conjugate extends to cancer cells, effectively reducing viability while the carrier itself remains mostly non-toxic. Conversely, as seen in Figures S5 and S6, the non-cancerous HaCaT cells demonstrated a higher tolerance to the treatment, suggesting a favorable selectivity profile for the formulation. At 24 h, the lowest concentration (50 µg/mL) showed no statistically significant toxicity compared to the controls. While higher doses (75–150 µg/mL) and prolonged exposure at 48 h resulted in a statistically significant decrease in survival (*p* < 0.05), the overall cytotoxic effect was less pronounced than that observed in the cancer cells (*p* < 0.001). Cell viability in the HaCaT line consistently remained above 54%, even at the maximum concentration of 150 µg/mL after 48 h. Taken together, these results indicate that the CNTs-PEG–Papiliocin construct exhibits a differential cytotoxic effect, reducing HeLa cell viability in a dose-dependent manner while having a statistically significant lower impact on the tested non-cancerous cell line.

### 2.6. Intracellular Delivery of Papiliocin via CNTs-PEG Induces Extrinsic Pathway Apoptosis in HeLa Cells

**Figure 7:**
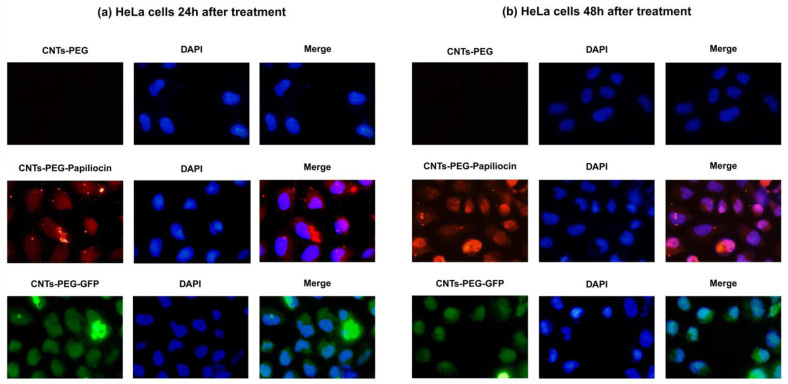
Intracellular uptake of functionalized CNTs (100 μg/mL) in HeLa cells (magnification 10×). Fluorescence microscopy images of HeLa cells incubated with functionalized carbon nanotubes at 24 h and 48 h. (Top Row) CNT-PEG (negative control) shows no autofluorescence. (Middle Row) CNTs-PEG–Papiliocin (therapeutic) shows distinct red fluorescence accumulating in both cytoplasm and nuclei, indicating successful delivery of the peptide. (Bottom Row) CNTs-PEG-GFP (quality treatment control) shows strong green fluorescence, confirming high-efficiency transport of macromolecules. (Note: While both GFP and Papiliocin formulations show high uptake, only the Papiliocin-loaded nanotubes induced apoptosis. Blue = DAPI).

Using immunofluorescence microscopy, we monitored the spatiotemporal trafficking of the CNTs-PEG–Papiliocin system in HeLa cells. At 24 h, the fluorescence signal exhibited a dispersed distribution, localizing to both the nuclear and cytoplasmic compartments (Figure 7, Middle Row, Panel a). At 48 h, a distinct shift in localization was observed, characterized by a predominant nuclear accumulation of the nanocomplexes (Figure 7, Middle Row, Panel b). The same applies to the quality control (CNTs-PEG-GFP). This migration contrasts with the vehicle control (CNTs-PEG), which showed no fluorescence.

Furthermore, Western blot analysis was performed to elucidate the apoptosis-mediated effect underlying the cytotoxicity observed in the MTT assay. Specifically, we evaluated the activation of key apoptotic markers—Caspase-8 and PARP-1—in HeLa cells treated for 24 and 48 h.

**Figure 8:**
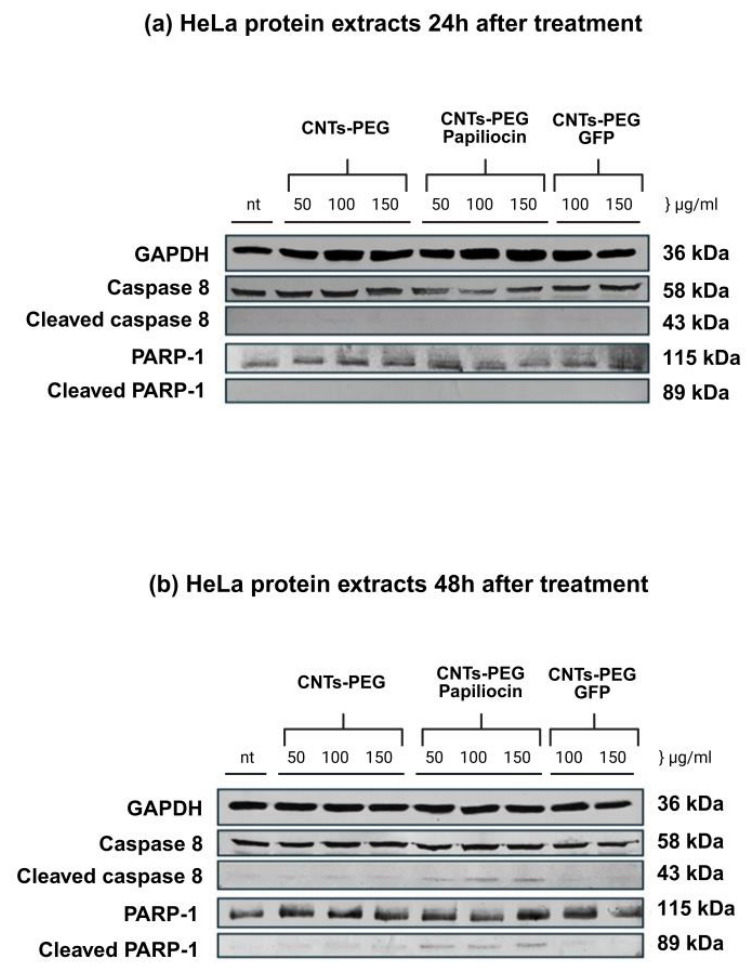
Western blot (WB) analysis of apoptotic markers in HeLa cells following treatment with CNTs-PEG, CNTs-PEG–Papiliocin, and CNTs-PEG-GFP nanocomplexes. Cells were treated with varying concentrations for 24 h (Top panel—(**a**)) and 48 h (Bottom panel—(**b**)). The expression levels of Pro-caspase 8, cleaved caspase 8, full-length PARP-1 and cleaved PARP-1 were examined. GAPDH was used as an internal loading control (nt stands for no treatment).

As shown in Figure 8 down below, the expression of the housekeeping protein GAPDH remained constant across all groups, ensuring equal protein loading (45 μg per lane). In the control groups treated with empty carrier (CNTs-PEG) and the quality control complex (CNTs-PEG-GFP), no activation of apoptotic proteins was observed, as evidenced by the lack of cleaved caspase bands and the stability of full-length PARP-1.

The activation of the extrinsic apoptotic pathway was indicated by the appearance of cleaved Caspase-8 (43 kDa), which was notably distinct after 48 h of treatment at higher concentrations (100 and 150 μg/mL). Most significantly, the cleavage of PARP-1, a hallmark of apoptosis, was clearly detected in the CNTs-PEG–Papiliocin-treated groups. While full-length PARP-1 (115 kDa) was present in all lanes, the specific apoptotic fragment, cleaved PARP-1 (89 kDa), appeared prominently in cells treated with the Papiliocin complex. This effect was not present at 24 h and became apparent at 48 h, particularly at concentrations of 100 and 150 mg/mL. These findings confirm that CNTs-PEG–Papiliocin induces cell death in HeLa cells primarily through a caspase-dependent apoptotic pathway. The kinetics of the apoptotic markers observed in the Western blot analysis strongly correlate with the subcellular trafficking profiles revealed by our immunofluorescence studies. As previously noted, the CNTs-PEG–Papiliocin complex exhibited a progressive nuclear accumulation, which was markedly profound at the 48 h timepoint.

**Figure 9:**
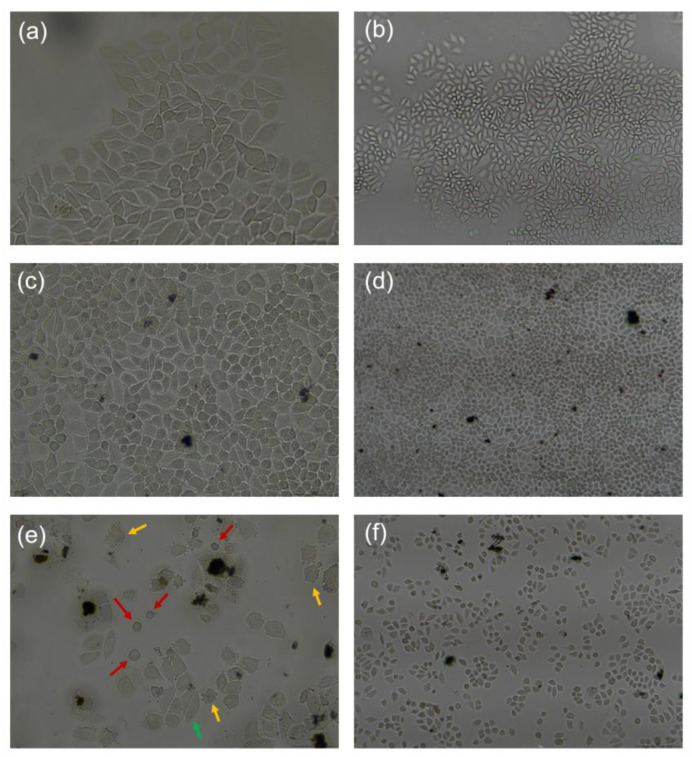
Morphological assessment of HeLa cells following treatment with CNT conjugates (100 μg/mL) for 48 h. (**a**,**b**) Untreated HeLa cells (Control) displaying characteristic polygonal morphology and forming a confluent monolayer ((**a**): 40×, (**b**): 10×). (**c**,**d**) HeLa cells incubated with CNTs-PEG; the cells maintain high density and typical spindle-like shapes ((**c**): 40×, (**d**): 10×). (**e**,**f**) HeLa cells treated with the high-loading CNTs-PEG–Papiliocin complex. In frame (**e**) (40×), distinct morphological hallmarks of cytotoxicity are observed: red arrows indicate cell shrinkage and rounding (indicative of apoptosis), yellow arrows highlight membrane blebbing and cytoplasmic granulation, and the green arrow marks a morphologically “normal” HeLa cell. Frame (**f**) (10×) demonstrates all the hallmarks mentioned above as well as a significant reduction in cell population and loss of confluency compared to the control and CNTs-PEG groups.

Inverted microscopy observations, as seen in Figure 9, further supported the MTT assay results and the apoptosis markers displayed by WB, revealing a significant, dose-dependent reduction in cell density in the CNTs-PEG–Papiliocin-treated groups compared to the confluent monolayers of the control groups. At 48 h, the treated population showed not only a marked decrease in total cell number but also a transition from a healthy, spindle-shaped morphology to a shrunken, rounded, and fragmented phenotype. This widespread loss of cell viability and the ‘shattered’ appearance of the remaining cells are consistent with the apoptotic program.

## 3. Discussion

The primary objective of this study was to evaluate the dual-action potential of Papiliocin–biofunctionalized carbon nanotubes (CNTs) as both a broad-spectrum antimicrobial agent and anticancer therapeutic. As detailed in the introduction, cationic antimicrobial peptides (AMPs) like Papiliocin target the anionic cellular membranes via electrostatic attraction, disrupting its integrity. Papiliocin was selected as the bioactive agent for this system due to its multi-faceted biological profile. Beyond its potent antibacterial activity, this peptide has demonstrated the ability to induce apoptosis in fungal cells while exhibiting negligible hemolytic effects and no cytotoxicity to non-cancerous mammalian cells [10,16]. Furthermore, Papiliocin offers significant anti-inflammatory and immunomodulatory properties, making it an ideal candidate for targeted biomedical applications [17]. However, clinical translation is often hindered by the peptides’ susceptibility to proteolytic degradation and low local concentration at the target site [19]. To address this, we immobilized Papiliocin onto PEGylated multi-walled carbon nanotubes. This delivery system was chosen for its high surface-area-to-volume ratio, which allows for high-density peptide loading, and its ability to penetrate complex biological barriers, such as bacterial membranes, biofilms and cellular membranes [24].

To determine the optimal bioactive threshold, we formulated two distinct conjugation ratios: a high-loading and a low-loading nanocomposite. This successful conjugation was confirmed by FT-IR and DLS/Zeta potential analysis in PBS. FT-IR spectra verified the peptide attachment, while Zeta potential measurements showed a shift toward neutral values as peptide loading increased. This charge neutralization in the PBS buffer led to the formation of larger hydrodynamic complexes, as seen in the DLS peak shifts. Our results demonstrated that immobilization density is a critical determinant of efficacy. The PEGylated vehicle (CNTs-PEG) showed low toxicity to both bacteria and mammalian cells, confirming that the biological activity is driven mostly by the conjugated peptide. Most importantly, the high-loading formulation significantly outperformed the low-loading formulation against both *E. coli* and *S. aureus*. This supports the hypothesis where a critical concentration of peptide is required to initiate membrane permeabilization events and, therefore, cytotoxicity. A defining feature of our results is the superior potency of the biofunctionalized CNTs against *E. coli* (Gram-negative) compared to *S. aureus* (Gram-positive). This observation aligns with the intrinsic molecular behavior of Papiliocin reported in structural studies. Kim et al. demonstrated that the N-terminal amphipathic helix of Papiliocin, specifically residues Trp^2^ and Phe^5^, possesses a high affinity for Lipopolysaccharide (LPS), the dominant outer membrane component of Gram-negative bacteria [10,14].

Beyond planktonic bacteria, biofilms represent a major clinical challenge due to the protective Extracellular Polymeric Substance (EPS) matrix, but AMPs seem to be a very promising combat tool towards them [31,32]. Our study revealed that while the CNTs-PEG control failed to disrupt *C. albicans* biofilms, the high-loading Papiliocin–CNT conjugate achieved a ~60% reduction in biofilm viability. This suggests that the biofunctionalized CNT scaffold mechanically pierces the EPS barrier to deliver the high-density peptide payload directly to the fungal cells. The inability of the low-loading formulation to achieve a determinable IC_50_ underscores that high peptide density is essential not just for cellular lysis, but for overcoming the diffusion barriers inherent to the biofilm matrix. Papiliocin is known for its ability to cause apoptosis to fungal cells [16].

Our study utilized HeLa cells to evaluate whether the Papiliocin–CNT system could re-activate cell death pathways. Even though cytotoxicity does not seem to be time-dependent, the apoptotic behavior of the Papiliocin–CNT hybrid system in cancer cells became apparent at 48 h, as shown through immunoblotting. Immunofluorescence analysis revealed a distinct spatiotemporal shift: the nanocomplexes initially localized both in the cytoplasm and nucleus in the first 24 h following incubation but accumulated profoundly in the nucleus by 48 h. This nuclear translocation likely triggers genotoxic stress. To elucidate the molecular mechanism, we analyzed key apoptotic markers. We found elevated expression levels of cleaved Caspase-8 and cleaved PARP-1 in HeLa cells treated with the high-loading conjugate, particularly at 48 h. This indicates the activation of the extrinsic apoptotic pathway where the Papiliocin–CNT hybrid acts as a death ligand [33]. The cleavage of PARP-1 (Asp214), a DNA repair enzyme, is a “point of no return” marker for apoptosis [34]. The absence of these markers in the CNTs-PEG and CNTs-PEG-GFP controls confirms that the apoptotic signaling is specific to the intracellular delivery of Papiliocin. Consistent with the findings of Kam et al. [35] regarding the transporter capabilities of carbon nanotubes, our data suggests that the internalization pathway leads to a critical accumulation within the nucleus, a step that is mandatory for the observed anticancer activity, while PARP cleavage suggests that the peptide nanocomplex directly interferes with nuclear stability. Cationic peptides can act as “anticancer peptides” (ACPs) by targeting the negatively charged membranes or binding to DNA once they bypass the plasma membrane [9,36]. Moreover, the CNTs-PEG–Papiliocin conjugate effectively triggers programmed cell death via apoptosis rather than uncontrolled necrosis; this is a highly desirable trait in drug development, as apoptotic pathways circumvent the pro-inflammatory signaling pathways typically associated with necrotic cell rupture [37,38].

Our findings align with emerging trends in nanomedicine, where high-aspect-ratio carriers like carbon nanotubes are used to enhance the therapeutic index of bioactive molecules [20,25,26,27,28]. While other groups have explored Papiliocin for infection control [14,15,16,17], the dual functionality observed here—where the same molecule acts as a membrane-lytic agent in bacteria and fungi and a pro-apoptotic nuclear agent in cancer cells—is significant. Various antimicrobial peptides (AMPs) have shown significant bioactivity when incorporated into hydrogels [39] and silver nanoparticles [40] and when used synergistically along with metal nanoparticles that possess antimicrobial properties [41]. The system presented here expands on these capabilities displayed by previous research work, offering a unique set of benefits such as extending the peptide’s half-life and its targeted delivery, while elucidating Papiliocin’s role as an anticancer agent. As stated previously and evidenced by immunofluorescence imaging, the immobilized peptide remains stable within the cellular environment even after 48 h incubation time, confirming that the conjugation sterically protects the peptide, avoiding rapid proteolytic degradation. Notably, the use of carbon nanotubes facilitates nuclear translocation of the peptide, likely mediated by the characteristic “nanoneedle effect,” which allows the tubes to penetrate cellular membranes and deliver the cargo directly into the nucleus [42]. Although the apoptotic markers observed via immunostaining and the inverted microscopy images display key apoptotic characteristics, further investigation is needed to completely elucidate Papiliocin’s apoptotic behavior. Another area for further optimization concerns selectivity. While the high-loading formulation demonstrates a therapeutic advantage—reducing HeLa cell viability by approximately 25% more than the non-cancerous HaCaT cells at the highest concentration—the observed impact on healthy cells, although encouraging, indicates that the selectivity profile is not yet ideal. Furthermore, the near-neutral surface charge of the complexes in PBS, as shown by our DLS and Zeta potential data, likely promotes some degree of aggregation that could influence non-specific interactions. Future efforts should also focus on fine-tuning the surface chemistry, perhaps, through more robust PEGylation or other PEG-modified analogs to overcome the reduction in the functional sites that can occur when using a homobifunctional polymer, in order to increase protein loading. Future investigations utilizing modified Papiliocin analogs with pronounced biological properties are expected to further optimize the complex’s efficacy and establish its therapeutic potential.

In conclusion, our results indicate that the Papiliocin–biofunctionalized CNT system could serve as a potent, density-dependent bioactive platform. The high-loading formulation provides the necessary multivalency to penetrate biofilms and bacterial membranes while possessing the intracellular stability to traffic to the nucleus and induce apoptosis in cancer cells.

## 4. Materials and Methods

### 4.1. Materials

Restriction endonucleases, T4 DNA ligase and Vent™ polymerase were purchased from New England BioLabs, Inc., Hitchin, UK. Molecular weight DNA and protein markers were obtained from Nippon Genetics (Nippon Genetics Europe GmbH, Düren, Germany). PCR primers for the cloning of Papiliocin were obtained from MWG, Ebersberg, Germany. pET-29c(+) was obtained from Novagen (Novagen, Madison, WI, USA). All other chemicals were obtained from Sigma–Aldrich Chemie GmbH, Steinheim, Germany.

### 4.2. Microorganisms and Growth Conditions

*Escherichia coli* (*E. coli*) and *Staphylococcus aureus* (*S. aureus*) were grown at 37 °C in Luria–Bertani (LB) medium and Terrific broth (TB) medium when needed, under vigorous shaking. *Candida albicans* (*C. albicans*), full-growth cultures were specifically prepared using 1X YNB (Yeast Nitrogen Base) medium under vigorous shaking at 30 °C for 3 days. Kanamycin and chloramphenicol were added in the culture media, when necessary, at final concentrations of 50 μg/mL and 34 μg/mL, respectively. The induction of recombinant Papiliocin expression in *E. coli* Rosetta [DE3] (Molecular Cloning Laboratories (MCLABs), San Francisco, CA, USA) carrying the expression plasmid pET29c–*Papiliocin*, was achieved by IPTG addition (0.5 mM) to the cultures when the absorbance at 600 nm was 0.8.

### 4.3. Plasmids

For the construction of the expression vector of recombinant Papiliocin fused to a C-terminal His_12_-tag, the corresponding DNA sequence was synthesized and cloned in pEX-K268 plasmid by Eurofins Genomics (Eurofins Genomics, Louisville, KY, USA). This plasmid (pEX–*Papiliocin*) was used as a template in a subsequent PCR. The primer pairs used consisted of the oligonucleotides 5′ CATATGCGCTGGAAAATTTTTAAA 3′ and 5′ AAGCTTCTATTAATGGTGATGGT 3′, comprising the NdeI and HindIII restriction sites, respectively. The PCR product was finally cloned into pET-29c expression vector (Novagen).

### 4.4. Purification of Recombinant Papiliocin

Transformed *E. coli* Rosetta [DE3] cells carrying pET29c-*Papiliocin* were grown at 37 °C in Terrific broth containing 50 μg/mL kanamycin and 34 μg/mL chloramphenicol induced with 0.5 mM IPTG. Following harvest, cells were lysed via sonication, and proteins were purified with affinity chromatography using Protino Ni-TED resin columns (Macherey–Nagel, Düren, Germany). During isolation, samples from the soluble fraction of cells (cytoplasmic fraction) were analyzed by SDS-PAGE and silver nitrate staining.

### 4.5. Electrophoresis, Western Blotting and Cell Lysis

SDS–polyacrylamide gel electrophoresis (SDS-PAGE) was performed using 10% or 15% (*w*/*v*) polyacrylamide gels containing 0.1% *w*/*v* SDS [43]. Protein concentrations were determined by the Bradford method [44], using bovine serum albumin as the reference standard. Proteins were stained with silver nitrate [45], electrotransferred [42] to nitrocellulose membranes (Macherey–Nagel, Düren, Germany) and immunostained, as previously described [46]. The primary antibodies used are illustrated below: monoclonal mouse His-tag, rabbit polyclonal Caspase-8 and rabbit monoclonal PARP. All were obtained from Cell Signaling Technology, except for the mouse monoclonal GAPDH antibody, which was purchased from Calbiochem (Merck KGaA, Darmstadt, Germany). Appropriate goat anti-mouse (Cell Signaling Technology, Danvers, MA, USA) and anti-rabbit (Cell Signaling Technology, Danvers, MA, USA) IgG AP-conjugated secondary antibodies were incubated with the membrane in 5% *w*/*v* non-fat skimmed milk for 90 min at room temperature. After washing the membrane with PBS-Tween 0.04% *v*/*v*, bands were developed in Alkaline Phosphatase Buffer by adding 50 mg/mL BCIP (Biotium, Fremont, CA, USA) and 50 mg/mL NBT (Biotium, Fremont, CA, USA) solutions. 

Hela cells incubated with different concentrations of CNTs-PEG (control sample), CNTs-PEG-GFP (quality control sample) and CNTs-PEG–Papiliocin at 24 h and 48 h, grown at a confluency of 70–80% in 12-well plates, were washed twice in ice-cold phosphate-buffered saline (PBS), lysed using an appropriate volume of RIPA Lysis Buffer (50 mM Tris-HCl pH 7.4, 150 mM NaCl, 1% *v*/*v* Triton X-100, and 1 mM PMSF) depending on cell population, and harvested from culture dishes on ice using a cell scraper. After being passed through a 25-gauge syringe needle eight to ten times, the whole extract was heated at 95 °C for 2 min. Whole-cell extracts were centrifuged for 10 min at 10,000× *g* at 4 °C in a microcentrifuge for efficient clarification, then separated at 45 µg of total protein lysates per well on 10% *w*/*v* SDS-PAGE, and transferred to nitrocellulose membrane for Western blot analysis, as mentioned above. The total protein concentration was calculated using the Bradford Assay.

### 4.6. Functionalization of CNTs with Bis(3-aminopropyl)Polyethylene Glycol

Oxidized CNTs (9.5 nm diameter × 1.5 μm length, Sigma–Aldrich Chemie GmbH, Steinheim, Germany) were firstly subjected to sonication, then carboxyl groups were activated by EDC/NHS, and finally bis(3-aminopropyl)PEG (MW 1500) was added. The now functionalized CNTs were filtered using 0.45 μm polycarbonate filters (Millipore, Burlington, MA, USA) and washed with water to remove excess reagents, followed by freeze-drying to obtain purified PEG-CNTs [25].

### 4.7. Immobilization of Papiliocin onto the CNTs-PEG Vehicles

For the protein conjugation of the surface of PEGylated CNTs, Papiliocin was added to the mixture at a concentration of 0.67 mg/mL and 2 mg/mL each to the CNTs-PEG dispersion (2 mg) to produce a low-loading and a high-loading formula of CNTs-PEG–Papiliocin, respectively. The same procedure was followed as previously described in Section 4.6.

### 4.8. Fourier Transform Infrared (FTIR) Spectroscopy

The chemical structure of the immobilized proteins onto CNTs was studied by recording their FTIR spectra (Perkin–Elmer, Spectrum One, Akron, OH, USA). The samples were made into pellets with potassium bromide, and a total of 64 spectra were averaged to reduce noise. All spectra were recorded at a resolution of 4 cm^−1^, and the recorded wavenumber range was 800–4000 cm^−1^.

### 4.9. Dynamic Light Scattering and Zeta Potential Studies

The average particle size (hydrodynamic diameter) and the surface charge of the PEGylated CNTs and the biofunctionalized PEGylated CNTs were determined via Dynamic Light Scattering (DLS) and Zeta potential measurements, respectively, using a Zetasizer Nano ZS (Malvern Instruments, Malvern, Worcestershire, UK). Prior to analysis, the loaded and unloaded nanotubes were dispersed in PBS 1X at a concentration of approximately 50 μg/mL. The Zeta potential was calculated from the electrophoretic mobility based on the Smoluchowski equation.

### 4.10. Antimicrobial Assay

The in vitro antibacterial activity of CNTs-PEG–Papiliocin towards *E. coli* and *S. aureus* bacterial strains was evaluated utilizing MMS (Minimal Mineral Salts) broth containing the concentrations of 150, 100, 75 and 50 μg/mL (dissolved in PBS) of the biofunctionalized CNTs-PEG–Papiliocin (low loading and high loading), using the CNTs-PEG vehicle as the control sample. The growth of bacteria was estimated by measuring the absorbance at 600 nm. The two cultivation media used for antibacterial activity tests were: (i) the Luria–Bertani broth with 1% *w*/*v* tryptone, 0.5% *w*/*v* NaCl and 0.5% *w*/*v* yeast extract; and (ii) the minimal medium salt broth containing 1.5% *w*/*v* glucose, 0.5% *w*/*v* NH_4_Cl, 0.5% *w*/*v* K_2_HPO_4_, 0.1% *w*/*v* NaCl, 0.01% *w*/*v* MgSO_4_·7H_2_O. The pH of the media was adjusted to 7.0.

### 4.11. Biofilm Formation Assay Using the TCP Method

Biofilm formation was assessed using the Tissue Culture Plate (TCP) method according to the following procedure: A volume of 0.5 mL of the bacterial/fungal suspension was transferred into each well of a 24-well along with the CNTs-PEG and CNTs-PEG–Papiliocin (low- and high-loading samples). The plates were incubated at 30 °C for 48 h under aerobic conditions. Following incubation, the supernatant containing planktonic cells was carefully removed via pipetting. The remaining biofilms were washed three times with 0.5 mL of 1X phosphate-buffered saline (PBS) to remove non-adherent cells. The biofilms were fixed by adding 0.5 mL of absolute methanol to each well for 20 min to ensure cell adherence to the plate surface. After discarding the methanol, the wells were stained with 0.1% *w*/*v* Crystal Violet solution for 20 min. Excess dye was removed by washing the wells with 0.5 mL of distilled water under gentle agitation. Subsequently, 33% *v*/*v* glacial acetic acid was added to each well and agitated gently to extract the dye from the biomass. The resulting solution was transferred to microcentrifuge tubes. The optical density (OD) was measured at 490 nm using a JENWAY 6305 UV/Vis Spectrophotometer (Thermo Fischer Scientific, Loughborough, UK) to quantify the total biofilm biomass [47].

### 4.12. Cell Culture

Human carcinoma HeLa and HaCaT keratinocyte cell lines were obtained from ATCC (American Type Culture Collection, Manassas, VA, USA) and was cultivated in DMEM containing 4.5 g/L glucose with L-glutamine and sodium pyruvate (Fischer, Gibco, Waltham, MA, USA), 10% *v*/*v* fetal bovine serum, and 1% *v*/*v* penicillin/streptomycin. The cells were maintained at 37 °C in a humidified 5% *v*/*v* CO_2_.

### 4.13. Immunofluorescence

HeLa cells were grown on glass coverslips. After the incubation period with different samples of CNTs (all 100 μg/mL), the cell coverslips were fixed with 4% w/v paraformaldehyde in PBS at room temperature. Following fixation, samples underwent wash with PBS. Cells were then quenched using a solution of 1 mL Tris-HCl (pH 7.5) and 9 mL PBS for 5 min. Cells were permeabilized with 0.2% v/v Triton X-100 in 1X PBS, followed by a single wash with 1X PBS. Non-specific binding sites were blocked using 0.5% w/v gelatin in 1X PBS and were incubated with the primary antibody (overnight at 4 °C). Post-incubation, the samples were washed with 0.1% v/v Triton X-100 in 1X PBS. Subsequently, samples were incubated with the secondary antibody for 1 h, followed by washes with 0.1% v/v Triton X-100 in PBS 1X and additional washes with 1X PBS. For nuclear visualization, samples were treated with DAPI (1:1000). Samples were then mounted for microscopic analysis, visualized in a Nikon confocal microscope using EZ-C1 3.20 software (Nikon Inc., Melville, NY, USA).

### 4.14. MTT Assay

A total of 5 × 10^3^ cells (HeLa and HaCaT) were seeded out in 96-well dishes using DMEM high glucose with 10% *v*/*v* FBS. After 24 h maintenance, the medium was switched with a fresh one in conjunction with different concentrations of the samples mentioned above. The viability of cultured cells was estimated by a 3-(4,5-imethylthiazol-2-yl)-2,5-diphenyltetrazolium bromide (MTT) metabolic assay at two different timepoints (24 h and 48 h).

### 4.15. Statistical Analysis and Visualization

Two-way ANOVA test was used for checking the differences between the distinct groups, with the statistical significance set at *p* = 0.05. The proper depiction with the corresponding statistical analysis was prepared using GraphPad Prism (Version 8.0.1) (https://www.graphpad.com/). ImageJ.JS (Version 1.54s) was used for merging and editing IF photos and OriginPro 2026 was utilized for FT-IR, DLS and zeta potential graphs.

## Data Availability

The experimental data are available upon request.

## Supplementary Materials

The following supporting information can be downloaded at https://www.mdpi.com/article/10.3390/molecules31101715/s1, Figure S1: Electrophoretic analysis of the *Papiliocin* gene and recombinant pET-29c construct; Figure S2: Expression and purification and of recombinant Papiliocin; Figure S3: Intensity-weighted grain size distribution of PEG-functionalized carbon nanotubes (CNTs-PEG) before and after Papiliocin loading, obtained via Dynamic Light Scattering (DLS); Figure S4: Zeta potential distribution profiles for (a) pristine CNTs-PEG, (b) low-loading Papiliocin formula, and (c) high-loading Papiliocin formula; Figure S5: In vitro cytotoxicity of CNTs-PEG, CNTs-PEG-GFP and CNTs-PEG-Papiliocin (high-loading formula) on HaCaT cells determined by MTT assay. The cells were treated with various concentrations (0–150) for 24 h; Figure S6: In vitro cytotoxicity of CNTs-PEG, CNTs-PEG-GFP and CNT-PEG–Papiliocin (high-loading formula) on HaCaT cells determined by MTT assay. The cells were treated with various concentrations (0–150) for 48 h.
