# Supplementary material for: Antimicrobial Peptide Papiliocin–Carbon Nanotube Hybrids: Potential Dual-Action Agents for Antimicrobial Activity and Apoptotic Cancer Cell Death
Source: Molecules. 2026 May 18;31(10):1715. doi: 10.3390/molecules31101715 (PMC13209427; doi:10.3390/molecules31101715)
Supplement: Supplementary file 1 [file molecules-31-01715-s001.zip › molecules-4245781-supplementary.pdf]

Supplementary Materials

Antimicrobial Peptide Papiliocin-Carbon Nanotube Hybrids: Potential Dual-Action Agents for Antimicrobial Activity and Apoptotic Cancer Cell Death

Konstantinos Zacheilas[1], Myrto Margariti[1], Maria Apostolia Pissia[2] and Rigini M. Papi[1,*]

[1] Laboratory of Biochemistry, Department of Chemistry, Aristotle University of Thessaloniki, University Campus, 54124 Thessaloniki, Greece
[2] Laboratory of Food Chemistry and Technology, Department of Chemistry, Aristotle University of Thessaloniki, University Campus, 54124 Thessaloniki, Greece
*Correspondence: rigini@chem.auth.gr

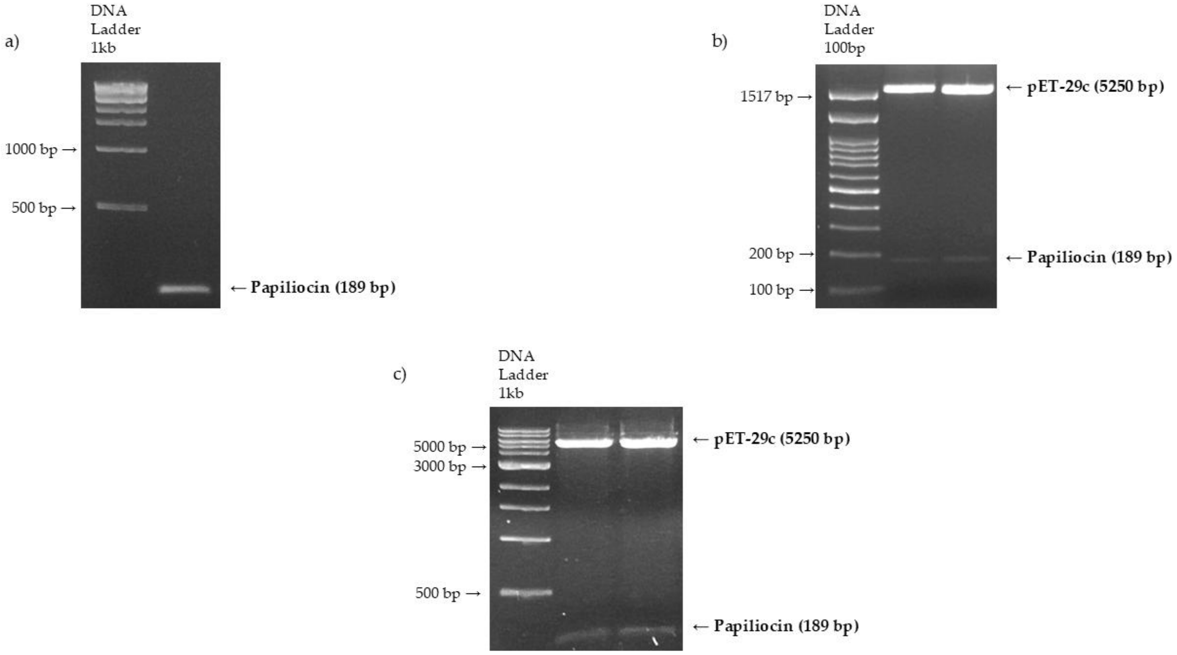

Figure S1. Electrophoretic analysis of the *papiliocin* gene and recombinant pET-29c construct. (a) PCR amplification of the papiliocin gene (189 bp) visualized on a 2% agarose gel against a 1 kb DNA ladder; the presence of a single, distinct band confirms successful and specific amplification of the target sequence. (b) Restriction digestion analysis of the recombinant plasmid using a 100 bp DNA ladder for high-resolution sizing of the lower molecular weight fragments. The upper bands represent the linearized pET-29c vector (5250 bp), while the lower bands confirm the excision of the papiliocin insert (189 bp) following double digestion. (c) Large-scale verification of the recombinant construct alongside a 1 kb DNA ladder; the

migration patterns of the vector backbone and the 189 bp insert are consistent with the theoretical molecular weights, confirming successful ligation and preparation of the expression system.

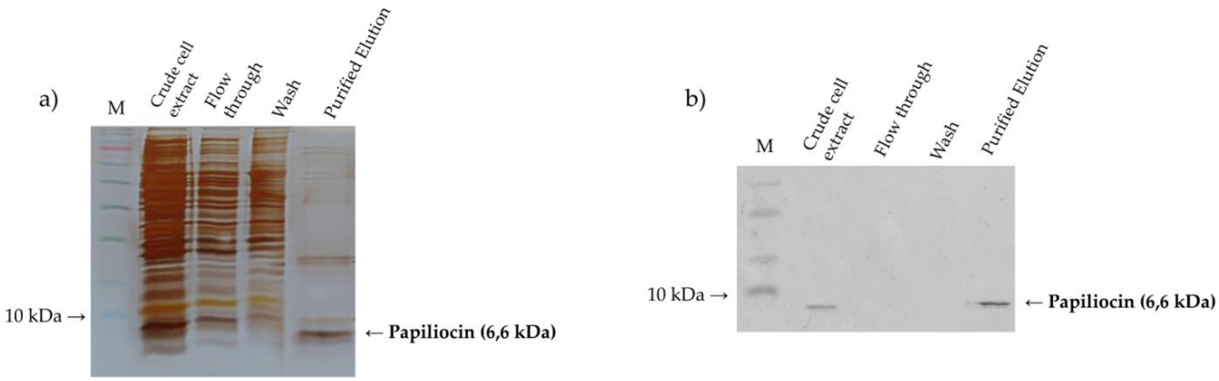

Figure S2. Expression and purification and of recombinant papiliocin. (a) SDS-PAGE analysis (silver nitrate-stained) showing the stages of protein purification from *E. coli* Rosetta (D3) cells after overexpression. Lane M: Molecular weight marker (10 kDa indicated); Crude cell extract: Total protein profile after cell lysis; Flow through and Wash: Removal of non-specific proteins during affinity chromatography; Purified Elution: Fraction showing a prominent band at approximately 6.6 kDa, corresponding to the predicted molecular weight of recombinant papiliocin. (b) Western blot analysis for specific detection of the target peptide. The protein was transferred to a membrane and probed with an anti-His-tag antibody, confirming successful overexpression and purification of the Papiliocin.

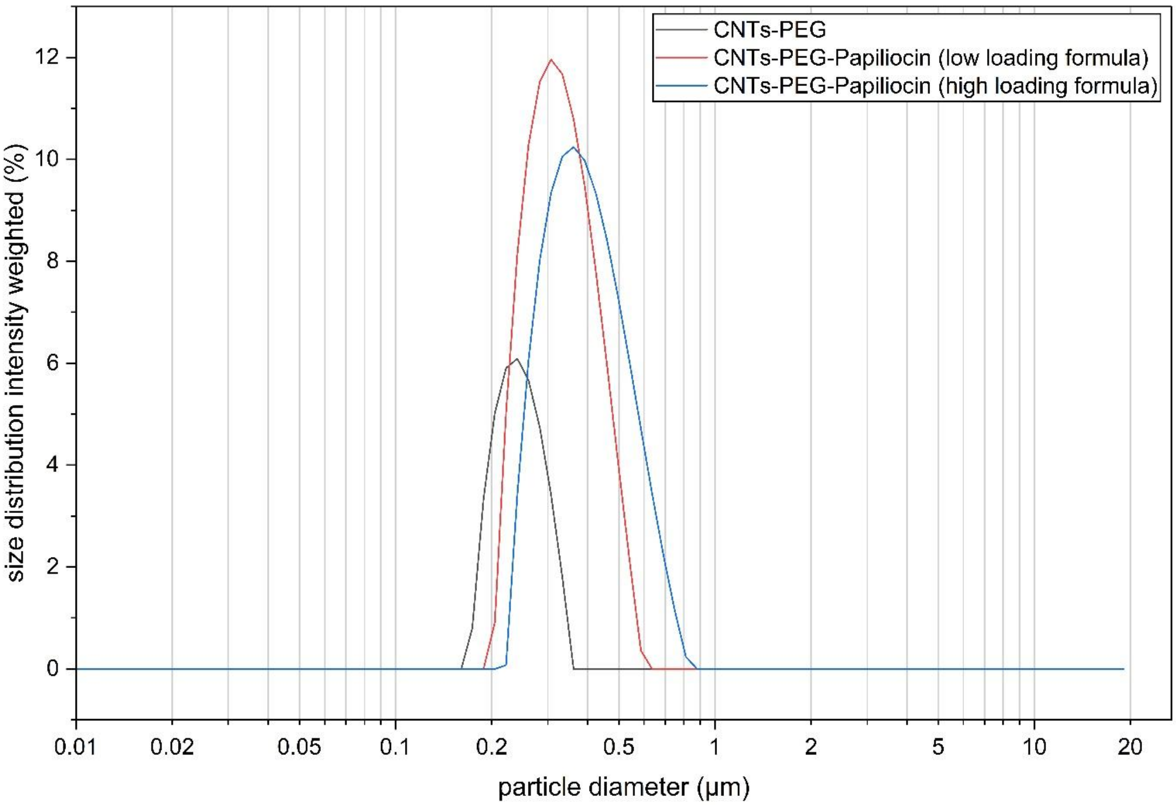

Figure S3. Intensity-weighted grain size distribution of PEG-functionalized carbon nanotubes (CNTs-PEG) before and after Papiliocin loading, obtained via Dynamic Light Scattering (DLS).

The hydrodynamic behavior of the functionalized carbon nanotubes was evaluated using Dynamic Light Scattering (DLS) to assess the impact of Papiliocin loading on particle size distribution. As illustrated in the intensity-weighted distribution plot, the CNTs-PEG base carrier (black line) exhibits a primary peak centered at approximately 220–250 nm. Upon the incorporation of Papiliocin, there is a clear concentration-dependent shift toward larger hydrodynamic diameters. The low loading formula (red line) shifts the peak to approximately 300 nm, while the high loading formula (blue line) further displaces the distribution, with the peak centering at approximately 350 nm. This progressive shift provides physical evidence of the peptide's accumulation on the nanotube surface, increasing the overall hydrodynamic volume of the complexes. The Polydispersity Index (PDI) values further characterize the physical state of the suspensions. CNTs-PEG exhibited a PDI of 0.427, reflecting the inherent heterogeneity and tendency of carbon nanotubes to form bundles. For the low loading formula, the PDI decreased to 0.352 and for the high loading formula, the PDI is 0.400.

Overall, the PDI values (ranging from 0.35 to 0.43) confirm that the samples are polydisperse systems. This is typical for high-aspect-ratio carbon nanotubes, as their non-spherical geometry and tendency to entangle prevent a strictly monodisperse distribution. The results confirm that while the peptide successfully coats the nanotubes, it also influences the overall particle size.

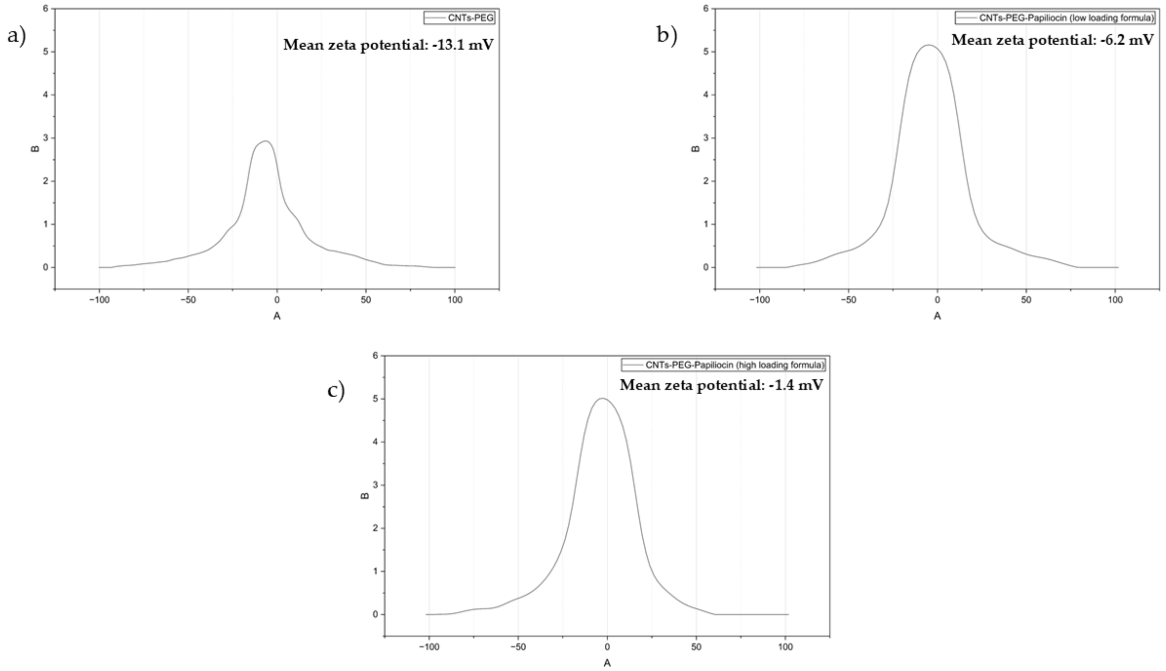

Figure S4. Zeta potential distribution profiles for (a) pristine CNTs-PEG, (b) low loading Papiliocin formula, and (c) high loading Papiliocin formula. Note the progressive shift in peak diameter and the neutralization of surface charge toward the isoelectric point as peptide concentration increases.

Zeta potential measurements provided further evidence of the electrostatic interaction between the carrier and the cargo. The pristine CNTs-PEG displayed a negative surface charge of -13.1 mV, likely due to residual oxygenated groups and the PEG functionalization. As the cationic (positively charged) Papiliocin was added, the surface charge was progressively neutralized. The mean zeta potential increased to -6.2 mV for the low loading formula and reached a near-neutral -1.4 mV for the high loading formula. This reduction in electrostatic repulsion (approaching the isoelectric point) directly supports the DLS findings; as the nanotubes lose their negative charge "shield," they are more prone to forming larger structures, resulting in the observed shift toward higher hydrodynamic diameters and the breadth of the size distribution peaks.

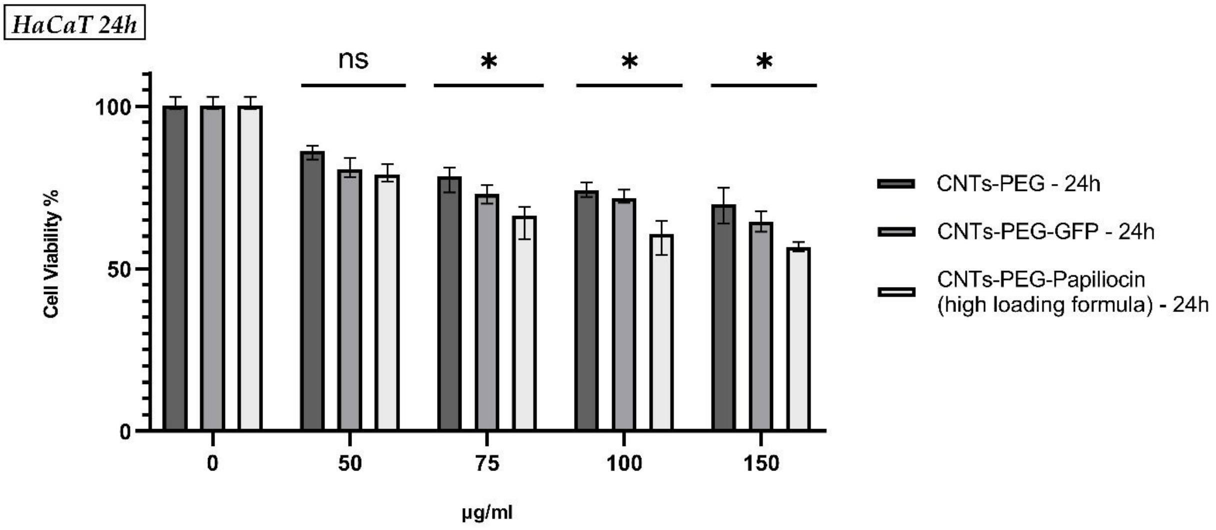

Figure S5. *In vitro* cytotoxicity of CNTs-PEG, CNTs-PEG-GFP and CNTs-PEG-Papiliocin (high loading formula) on HaCaT cells determined by MTT assay. The cells were treated with various concentrations (0 – 150) for 24 h. Statistical analysis was performed using GraphPad Prism software (Version 8.0.1) (GraphPad, San Diego, CA, USA) Statistical significance was determined using the p-value ($p < 0.05$ (represented by *)).

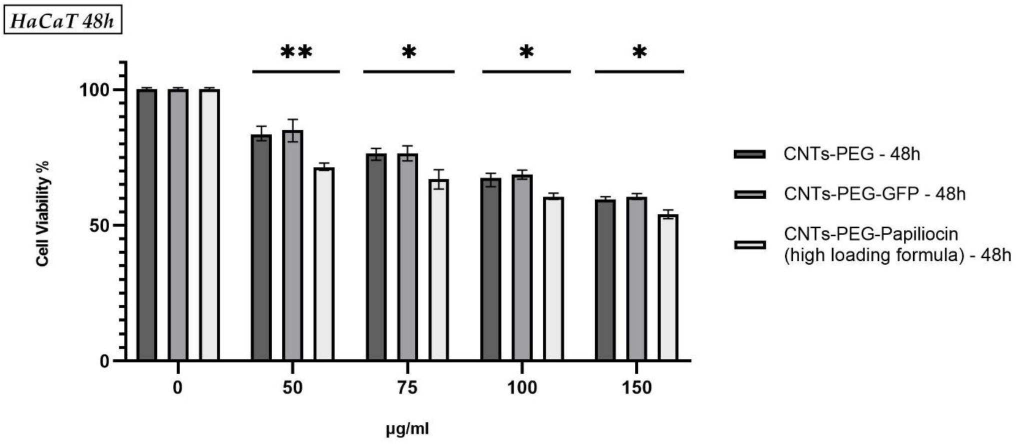

Figure S6. *In vitro* cytotoxicity of CNTs-PEG, CNTs-PEG-GFP and CNTs-PEG-Papiliocin (high loading formula) on HaCaT cells determined by MTT assay. The cells were treated with various concentrations (0 – 150) for 48 h. Statistical analysis was performed using GraphPad Prism software (Version 8.0.1) (GraphPad, San Diego, CA, USA) Statistical significance was determined using the p-value ($p < 0.05$ (represented by * and p-value < 0.01 represented by **)).
